# Development of a portable label-free electrochemical sensor modified with AuNPs/g-C_3_N_4_@CTAB for uric acid detection in complex blood samples

**DOI:** 10.1007/s00604-026-08027-1

**Published:** 2026-04-24

**Authors:** Fernando C. Gallina, Igor. G. S. Oliveira, Muriel A. Rodrigues, Luís F. Basso, Oswaldo C. Junior, Beatriz T. Marin, Robson S. Souto, Leandro M. C. Pinto, Herintha C. Neitzke-Abreu, Thalles P. Lisboa, Marcos R. V. Lanza, Willyam R. P. Barros

**Affiliations:** 1 Faculty of Exact Sciences and Technology, Federal University of Grande Dourados – UFGD, Rodovia Dourados-Itahum, km 12, Dourados, MS 79804˗970 Brazil; 2 São Carlos Institute of Chemistry, University of São Paulo, Av. João Dagnone, 1100, São Carlos, SP 13563˗120 Brazil; 3 Institute of Chemistry, Federal University of Mato Grosso do Sul, Av. Costa e Silva, s/nº, Campo Grande, MS 79070-900 Brazil; 4 Faculty of Health Sciences, Federal University of Grande Dourados – UFGD, Rodovia Dourados-Itahum, km 12, Dourados, MS 79804˗970 Brazil

**Keywords:** Graphitic carbon nitride, Cetyltrimethylammonium bromide, Clinical sample, Electrochemical platform, Differential pulse voltammetry, Screen-printed electrode

## Abstract

The development and application of a lab-made screen-printed electrode (SPE) modified with electrodeposited gold nanoparticles (AuNPs), graphitic carbon nitride (g-C_3_N_4_) and cetyltrimethylammonium bromide (CTAB) for the electrochemical determination of uric acid (UA) is reported. The SPE/AuNPs/g-C_3_N_4_@CTAB platform was optimized to enhance its electroactive surface area, conductivity, and charge-transfer kinetics, as confirmed through scanning electronic microscopy, energy-dispersive x-ray spectroscopy, Raman spectroscopy, contact angle analysis, cyclic voltammetry, and electrochemical impedance spectroscopy. Differential pulse voltammetry measurements demonstrated a linear response for UA concentrations between 50 and 600 µmol L⁻¹, with a limit of detection of 1.95 µmol L⁻¹, fully covering clinically relevant ranges. The sensor exhibited high selectivity against common interferents, as well as excellent stability and optimal performance at physiological pH of 7.0, consistent with UA dissociation equilibria. The application of the SPE/AuNPs/g-C_3_N_4_@CTAB sensor for UA detection in human whole blood, serum, and plasma samples showed promising results comparable to those obtained from standard enzymatic colorimetric assays, confirming the analytical reliability of the device. The computational analysis by DFT calculations demonstrated that the *E*_*ads*_ of UA to the complete modified sensor decreased at − 0.23 eV when compared to the SPE/AuNPs/g-C_3_N_4_. This reduction provides strong theoretical evidence supporting the influence of the CTAB modifier on the UA detection mechanism. Overall, the proposed modified SPE platform provides a simple, cost-effective, and portable alternative tool for UA monitoring in clinical samples.

**Graphical abstract:**

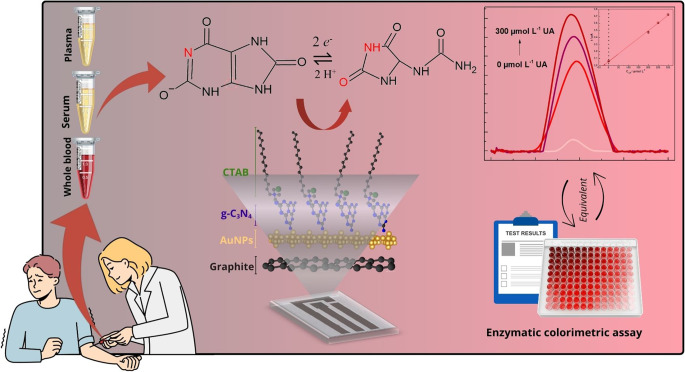

**Supplementary Information:**

The online version contains supplementary material available at 10.1007/s00604-026-08027-1.

## Introduction

Uric acid (UA) is a critical biomarker for human health in clinical contexts, which is normally detected in blood and urine samples and represents the final, poorly soluble product of purine degradation; the normal concentration of UA found in human serum roughly ranges from 178 to 416 µmol L^− 1^ for males and from 178 to 357 µmol L^− 1^ for females [1–4].

The medical condition characterized by the presence of elevated levels of UA in human blood is referred to as hyperuricemia; this condition is primarily associated with several health issues, including hypertension, ischemic heart disease, Lesch-Nyhan syndrome and gout, which is a result of monosodium urate formation and deposition in joints and soft tissues that leads to inflammatory responses [5–7]. Given the serious risks it poses to human health, UA determination and monitoring in biological samples are of extreme importance. When it comes to UA determination, electrochemical techniques, coupled with screen-printed electrodes (SPE), have emerged as a compelling alternative tool that offers key advantages, including lower sample volumes, high sensitivity, operational simplicity and ease of analysis, miniaturization capability for in-situ application and rapid results [3, 8].

Generally, electrochemical strategies for UA detection are divided into enzymatic biosensors and non-enzymatic sensors, with distinct advantages and limitations. Enzymatic biosensors, typically based on the immobilization of enzymes, and in the case of UA detection, uricase, offers high selectivity due to the specific recognition mechanism that the enzyme provides. However, their widespread commercial application is often hindered by the instability of enzymes, which are sensitive to environmental factors such as temperature, humidity, and pH, and require complex immobilization procedures, and suffer from high cost and limited shelf-life [9].

Conversely, non-enzymatic sensors, which rely on the direct electro-oxidation of UA at the electrode surface, have garnered significant attention for their cost-effectiveness, robust stability, and simple fabrication processes. Despite these operational benefits, non-enzymatic approaches using bare electrodes often face challenges, including high overpotentials, slow electron transfer kinetics, and susceptibility to surface fouling by oxidation products [10]. Consequently, the development of advanced electrode surface modifiers is essential to enhance the catalytic activity and selectivity of non-enzymatic sensors. The importance of sensor performance in measuring biological and non-biological analytes lies in their accuracy, low limit of detection (LOD), and rapid response, as these features enable real-time monitoring and reliable decision-making [11, 12].

As such, one underlying advantage of this electrode is the fact that it can easily be modified with a wide range of materials that have been extensively explored in literature [13]. In this context, the electrodeposition of Au nanoparticles (AuNPs) on the electrode surface has been found to contribute to an efficient electron transport through its high conductivity [14–17] and larger surface area, which play a key role in enhancing the electroactive sites [18, 19]. While bimetallic alloys offer synergistic catalytic enhancements, monometallic AuNPs were selected for this platform to prioritize methodology simplicity and cost-effectiveness without compromising stability [20]. Additionally, AuNPs are well-documented for their excellent conductivity, providing a reliable and sufficient electron transfer interface for UA oxidation when coupled with high-surface-area supports [21, 22], avoiding the complex optimization required for alloy-based systems [23, 24].

Graphitic carbon nitride (g-C_3_N_4_) has been employed as a modifier of SPE due to its easy synthesis process that involves safe and accessible precursors, such as melamine [25], as well as its environmentally friendly aspect, considering that it is a non-metal semiconductor. Additionally, of the polymorph forms of carbon nitride, g-C_3_N_4_ exhibits higher chemical and physical stability, high electron transfer rate and a higher number of surface-active sites [26]. However, one setback of g-C_3_N_4_ is its low specific surface area, which poses a problem when it comes to its application in electrochemical sensing. One way to overcome this problem is using an exfoliating process, in which sufficient energy is provided (*e.g.*: ultrasonic bath), thus breaking the weak van der Waals forces between g-C_3_N_4_ stacked layers, resulting in g-C_3_N_4_ nanosheets characterized by higher conductivity and larger specific surface area [27, 28].

Other methods that have been employed to further enhance the conductivity of g-C_3_N_4_ include elemental doping, modified composite formations and mixtures using metallic materials post g-C_3_N_4_ - synthesis [13, 26]. Furthermore, pre-synthesis methods have also been employed in combination with different solvents and compounds in order to ensure higher conductivity and better dispersion. In a recent study related by Mane et al. (2024), was incorporated cetyltrimethylammonium bromide (CTAB) into graphitic carbon nitride sheets which helped increase the electroactive area of the sensor [29].

The incorporation of CTAB into the sensing platform can be primarily beneficial to the electrode because of the suitable properties and characteristics of CTAB. As a quaternary ammonium cationic surfactant, CTAB consists of a positively charged polar hydrophilic head, especially when it comes into contact with water, and a long nonpolar hydrophobic tail that facilitates interaction with the electrode surface through its cationic group; these properties, when incorporated into the electrode, essentially helps to design an efficient sensing device that exhibits a higher number of electroactive sites, greater electron transfer rate, higher conductivity and excellent overall sensitivity [30, 31].

Taking the considerations above, the present study reports the development and application of a lab-made SPE based on graphite ink and modified with AuNPs/g-C_3_N_4_@CTAB for the electrochemical determination of UA. The electrochemical sensor exhibited excellent sensitivity and selectivity towards UA when applied for the analyte determination in human whole blood, serum, and plasma samples. Additionally, the optimized method was validated through a commercial technique (enzymatic colorimetric assay), where it showed statistically comparable results. The findings of the study provide useful insights into the effectiveness of the modifiers used in the AuNPs/g-C_3_N_4_@CTAB-based SPE, constructed as a simple, cost-effective and portable lab-made electrochemical device, which has proven to be suitable for application toward UA determination in biological matrices.

## Experimental

### SPE construction

The SPE was constructed and characterized according to the steps previously reported by our research group [15–17, 32, 33]. The graphite ink used in the construction of the SPE was constituted by a mixture of graphite powder (CAS 7782–42 − 5, Dinâmica, Brazil), stained glass varnish (Acrilex, Brazil), and acetone (CAS 67–64 − 1, LS Chemicals, Brazil). Using acetone as solvent, the carbonaceous material and polymeric resin were mixed in the ratio of 70%:30% (w/w) until a complete homogenization was obtained. The mixture was then applied on the surface of the polypropylene sheet using a paintbrush, resulting in the complete SPE, comprising of a tri-electrode system based on carbon materials, including counter and *pseudo* reference electrode, aiming a lower production cost, as well as less steps on SPE manufacturing, an approach that is already reported in the literature [34, 35].

### g-C_3_N_4_ synthesis and g-C_3_N_4_@CTAB dispersion

g-C_3_N_4_ synthesis was carried out by weighing 5.0 g of melamine (CAS 108-78−1, Merck, Brazil) in a crucible, which was calcinated at 550 °C in a muffle oven (ramp up of 5 °C min^− 1^) for 5 h. This process gave rise to a yellow powder that was left to cool at room temperature and was then finely ground. The g-C_3_N_4_ obtained was stored in an amber flask.

The g-C_3_N_4_@CTAB dispersion was obtained by mixing the synthesized g-C_3_N_4_ and CTAB (CAS 57-09−0, Merck, Brazil) in 2.0 mL (w/w); the procedures employed for the synthesis were as follows: (i) 0.5, 1.0, and, 2.0 mg mL^− 1^ of g-C_3_N_4_ was added to a 2.0 mL microtube filled up with ultrapure water; (ii) the solution was then ultrasonicated for 6 h (Unique USC-800, 40 kHz); and (iii) 1.0, 2.5, and 5.0 mmol L^− 1^ CTAB was added to the solution, which was further ultrasonicated for 30 min.

### SPE modification with AuNPs and g-C_3_N_4_@CTAB

Firstly, AuNPs electrodeposition was carried out by chronoamperometry and cyclic voltammetry (CV), where 0.5 mmol L^−1^ hydrogen tetrachloroaurate (HAuCl_4_⋅3H_2_O) (CAS 16961–25 − 4, Merck, Brazil) was applied in 0.1 mol L^−1^ phosphate buffer solution (PBS), at pH 7.0. The parameter conditions used in each technique are presented in Table S1 and the electrodeposition profiles can be found in Fig. S1 in Supplementary Information.

Subsequently, 10 µL of g-C_3_N_4_@CTAB was applied on the surface of the working electrode by drop-casting and dried at room temperature and the resulting electrochemical device was named SPE/AuNPs/g-C_3_N_4_@CTAB.

### Chemical and electrochemical characterization of the SPE/AuNPs/g-C_3_N_4_@CTAB

The Scanning Electron Microscopy (SEM) images were obtained from the microscope model JEOL-JSM 7200 F (Akishima, Japan), and the surface composition analysis was conducted by energy-dispersive X-ray spectroscopy (EDX) using the same equipment coupled with a Bruker XFLASH 6–60 (Massachusetts, USA), in a selected area of 36 μm². Contact angle measurements were carried out using an Ossila (Sheffield, UK) contact angle goniometer. The chemical structure of the SPE surface was determined through Raman spectroscopy analysis using a Dimension P2 spectrometer.

The electrical properties of the SPE surface was analyzed through the application of electrochemical impedance spectroscopy (EIS) on a Metrohm Autolab PGSTAT302N potentiostat/galvanostat using 0.1 mol L^− 1^ KCl as supporting electrolyte, in the presence of 1.0 mmol L^− 1^ [Fe(CN)_6_]^3−^/[Fe(CN)_6_]^4−^, in a frequency range of 0.1 Hz to 100 kHz (10 points per decade), with amplitude of 5 mV (r.m.s.) and applied potential of + 0.0374 V (vs. graphite pseudo reference electrode).

Cyclic voltammetry was carried out on a Metrohm Autolab PGSTAT302N potentiostat/galvanostat, using 0.1 mol L^− 1^ KCl as supporting electrolyte, in the presence of 1.0 mmol L^− 1^ [Fe(CN)_6_]^3−^/[Fe(CN)_6_]^4−^, in a potential window of −0.8 to + 0.8 V; this was done in order to further elucidate the effects of the modifiers on the electroactive surface area.

### Sample collection and preparation

Whole blood, plasma, and serum samples were obtained from male and female human subjects at the Health Science Research Laboratory - LPCS/UFGD. This study was conducted under CAAE registration number 79671224.4.0000.5160, and its protocol was approved under number 6.874.174.

Approximately 10 mL of venous blood was collected from the basilic vein. To prepare whole blood and plasma, a portion of each sample was collected in a tube containing EDTA. Plasma was then separated by centrifugation at 3.0 G for 10 min. To obtain serum, another portion of the blood was placed in a tube with a clot activator and centrifuged at 3.0 G for 10 min. The resulting serum was then aliquoted into 1.5 mL microtubes and stored at −20 °C until it could be analyzed.

### UA detection using the SPE/AuNPs/g-C_3_N_4_@CTAB

For the electroanalysis of UA, differential pulse voltammetry (DPV) and CV were carried out using 200 µmol L^− 1^ and 1 mmol L^− 1^ UA, respectively, in 0.1 mol L^− 1^ phosphate buffer solution (PBS) at pH 7.0, in a potential window of 0.0 to + 0.8 V for DPV and − 0.2 to + 1.0 V for CV. DPV parameters were optimized, and the final optimized parameters are presented in Table S2. All further studies were conducted using the optimized parameters.

pH was also optimized by testing in the range of 3.0 − 9.0 in 0.1 mol L^− 1^ PBS. The analytical curve was constructed in the UA concentration range of 50 to 600 µmol L^− 1^. Recovery analysis was conducted using whole blood, serum, and plasma samples from both male and female human. The samples were diluted 1:10 in 0.1 mol L⁻¹ PBS at pH 7.0, prior to analysis. Additionally, the same samples were analyzed by the Instituto Hermes Pardini laboratory (CNPJ: 19.378.769/0001–76) using established commercial enzymatic colorimetric assays to validate the performance of the SPE/AuNPs/g-C_3_N_4_@CTAB.

An interference study was performed using a 1:10 UA: interferent ratio. The potentially interfering compounds employed for the interference analysis were as follows: glucose (CAS 50–99−7, Merck, Brazil), urea (CAS 7–13−6, Merck, Brazil), dopamine (CAS 51–61−6, Sigma-Aldrich, USA), progesterone (CAS 57–83−0, Merck, Brazil), estriol (CAS 50-27−1, Merck, Brazil), ion sodium (CAS 7647-14−5, Êxodo Científica, Brazil), ion potassium (CAS 7447-40−7, Proquímios, Brazil), ion calcium (CAS 10043-52−4, Êxodo Científica, Brazil), ion magnesium (CAS 10034-99−8, Proquímios, Brazil), folic acid (CAS 59-30−3, Farmácia Possanga, Brazil), ascorbic acid (CAS 50–81−7, Merck, Brazil), and creatinine (CAS 60-27−5, Merck, Brazil). A memory effect study was conducted to analyze the electrochemical device stability through consecutive measurements on the same electrode, with increasing concentrations of UA, starting at 200 µmol L^− 1^ up to 350 µmol L^− 1^, and rolling the concentration back to 200 µmol L^− 1^.

### Investigation of the UA detection mechanism by computational methods

Density Functional Theory (DFT) calculations were performed using the GPAW code [36, 37] with the PBE functional [38, 39]. A plane-wave basis set with 450 eV cutoff energy and a 4 × 4 × 1 Monkhorst-Pack [40] k-point grid were employed. Geometry optimization was considered converged when forces fell below 0.02 eV/Å and energy changes between iterations were less than 10^− 5^ eV.

The lab-made SPE modified with electrodeposited AuNPs and g-C_3_N_4_@CTAB dispersion was modeled using a slab approach with a 15 Å vacuum layer. The graphite substrate was represented by a graphene-like layer, with AuNPs clusters simulating the electrodeposited nanoparticles. The g-C_3_N_4_@CTAB composite was positioned above the Au-modified surface, with CTAB’s cationic head groups oriented toward the interface.

UA adsorption was investigated by placing the molecule at various sites on the optimized surface, followed by structural relaxation. Adsorption energies (*E*_*ads*_) were calculated by Eq. 1, where *E*_*system+UA*_, *E*_*system*_, and *E*_*UA*,gas_ represent the total energies of the composite with adsorbed UA, the pristine surface, and isolated UA in the gas phase, respectively.

1 $$E_{ads}=E_{system+UA}-\left(E_{system}+E_{UA,\;gas}\right)$$

## Results and discussion

### Optimization of g-C_3_N_4_ and CTAB concentration

The optimum concentration of the modifiers applied for UA detection was analyzed by DPV using PBS, at pH 7.0, in the presence of 200 µmol L^− 1^ UA, as shown in Fig. S2A. Firstly, g-C_3_N_4_ concentration was analyzed by testing the concentrations of 0.5, 1.0, and 2.0 mg mL^− 1^, while CTAB concentration was fixed at 5 mmol L^− 1^ and AuNPs were electrodeposited by chronoamperometry (150 s, 0.5 mmol L^− 1^ HAuCl_4_⋅3H_2_O). The findings obtained demonstrate that the concentration level of g-C_3_N_4_ influences UA peak current (i_*p*_*)* response, where the lowest concentration yielded better electrochemical performance; this outcome can be attributed to the large structure of g-C_3_N_4_ heptazines and triazines [41], which may block the electrode surface, hindering UA-electrode interaction. In view of that, g-C_3_N_4_ concentration of 0.5 mg mL^− 1^ was chosen for the conduct of further studies.

CTAB optimization was performed using three different concentrations: 1, 2.5, and 5 mmol L^− 1^. Fig. S2B shows that the i_*p*_ response for UA is optimal at 2.5 mmol L^− 1^ of CTAB; this outcome indicates that higher or lower CTAB concentrations diminish the electrochemical performance of the sensing device. These findings are in agreement with surfactants behavior as well documented in the literature, where, at a certain threshold, known as the critical micelle concentration (CMC), surfactant monomers are aggregated, resulting in a structure where the hydrophilic head is pointed towards the solvent (“outside”), while the hydrophobic tails are pointed to the center of the micelle (“inside”) [31].

While the concentration of 2.5 mmol L⁻¹ exceeds the standard literature values for the CMC of CTAB (typically cited around 0.9–1.0 mmol L⁻¹ [42]. It should be noted, however, that the transition to a micellar phase can be shifted or suppressed by the complex interplay of thermodynamic factors, such as the hydrophobic tail, temperature, pH and electrolyte [43, 44]. Since the CMC is a thermodynamic threshold sensitive to the local environment, including electrolyte concentration and temperature, the nominal CMC value reported for pure water may not strictly apply to this multicomponent system.

A comparative analysis of the electrochemical response obtained for each concentration showed that both 1.0 mmol L^− 1^ and 2.5 mmol L^− 1^ concentrations may have formed micelles; this possibility will be further discussed in Section "Morphological and chemical characterization", since without the formation of micelles, the presence of free CTAB in the solution can also lead to an improvement in conductivity and dispersion through adsorption on the electrode surface [30, 31, 45].

As for the CTAB concentration of 5.0 mmol L^− 1^, higher concentrations of surfactants can create a layer on the electrode surface, which blocks the electrode/solution interface, hindering the electrochemical performance. In this sense, the CTAB concentration was set to 2.5 mmol L^− 1^.

### Optimization of AuNPs electrodeposition

The electrodeposition of AuNPs was first subjected to optimization analysis by comparing the following chronoamperometry times (see Table S1 for the parameters applied): 90, 120, 150, and 170 s (Fig. S3A). It was observed that 150 s proved to be the optimal application time for UA electrochemical response; this result shows that shorter application time does not allow enough AuNPs to be electrodeposited on the electrode surface, while longer application times may allow the deposition of enough AuNPs to block the electrode/solution interface. Thus, the chronoamperometry analysis time was set to 150 s.

The second optimization analysis on the electrodeposition of AuNPs was conducted by comparing two techniques: CV and chronoamperometry. Fig. S3B shows the increase in UA i_*p*_ value when AuNPs are electrodeposited by chronoamperometry, even though CV electrodeposition was successful, as indicated by its profile shown in Fig. S1B. These findings can be attributed to the fact that, due to its controlled electrodeposition potential, chronoamperometry promotes better control of nucleation, leading to the formation of more AuNPs and favoring a more homogeneous nanoparticle size and distribution. In contrast, CV is relatively more time consuming, and its consecutive cycles can lead to the creation of layers of AuNPs that block electroactive sites [46–48].

### Morphological and chemical characterization of the SPE/AuNPs/g-C_3_N_4_@CTAB

**Fig. 1:**
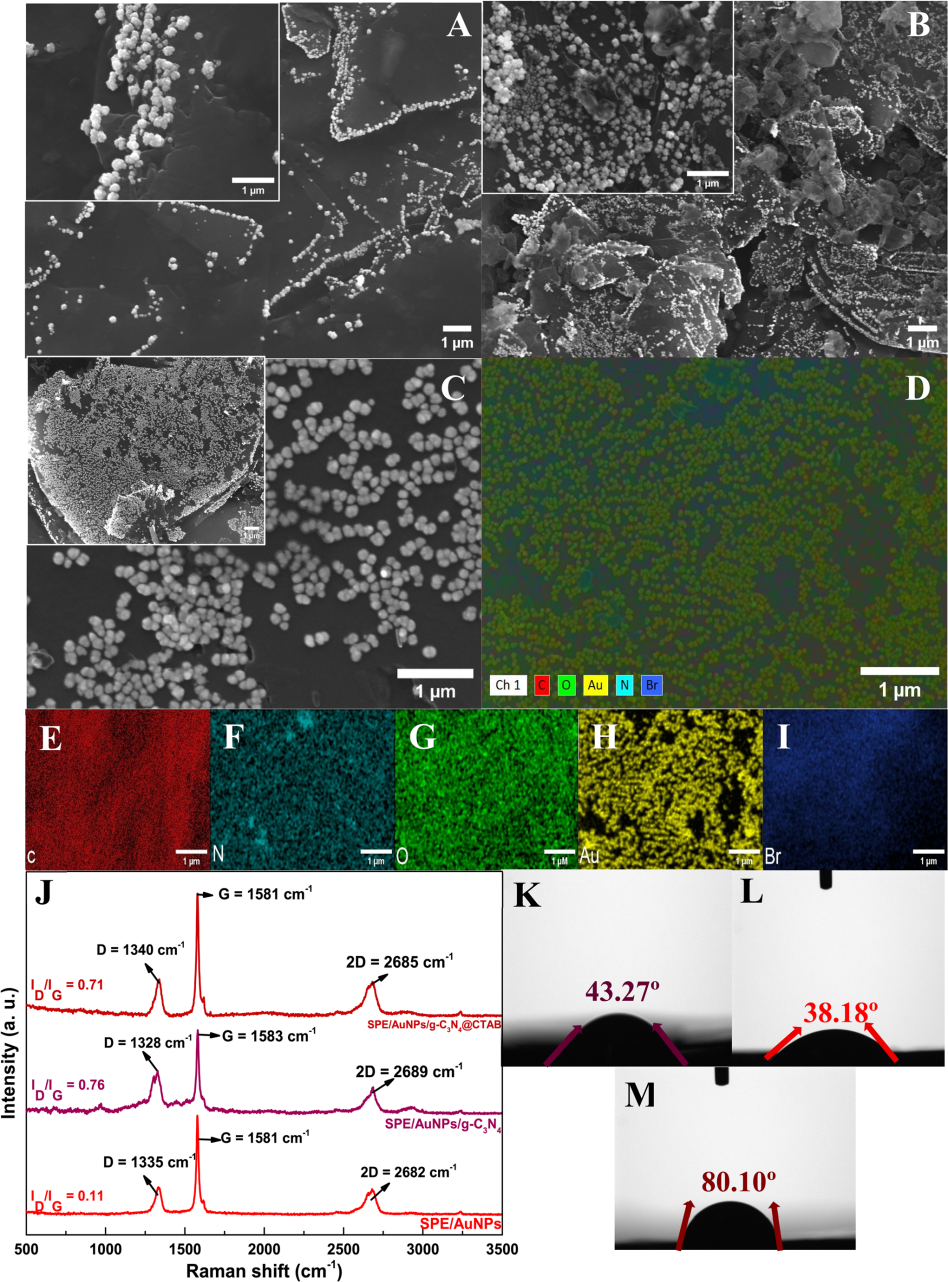
SEM images (**A** to **C**:) **A**) SPE/AuNPs magnitude of ×7500 and inset of×50000; **B**) SPE/AuNPs/g-C_3_N_4_ magnitude of ×7500 and inset of ×50000; **C)** SPE/AuNPs/g-C_3_N_4_@CTAB magnitude of ×7500 and inset of ×50000; EDS mapping (**D** to **I**): **D**) elemental mapping of the SPE/AuNPs/g-C_3_N_4_@CTAB; **E**) carbon; **F**) nitrogen; **G**) oxygen; **H**) Au and **I**) Bromide. **J**) Raman spectra of SPE, SPE/AuNPs/g-C_3_N_4_and SPE/AuNPs/g-C_3_N_4_@CTAB; Contact angle measurements: **K**) SPE/Au, **L**) SPE/AuNPs/g-C_3_N_4_ and **M**) SPE/AuNPs/g-C_3_N_4_@CTAB.

To further understand the effects of the modifiers on the SPE/AuNPs/g-C_3_N_4_@CTAB, characterization studies were carried out where the following elements were thoroughly evaluated: morphological structure, chemical composition, electrochemical behavior, and performance. Firstly, SEM images were acquired to help analyze the SPE surface morphology (Fig. 1A to C). In Fig. 1A, one can clearly see the irregular layered graphite flakes of the electrode surface, with AuNPs successfully electrodeposited on top in spherical cauli-flower -shaped clusters.

After drop-cast deposition of g-C_3_N_4_ (Fig. 1B), one can see agglomerated nanosheets adhered to the AuNPs layer, which is quite similar to what has been reported in the literature [49]. Finally, Fig. 1C shows the surface of the proposed sensor, where the g-C_3_N_4_@CTAB solution is deposited by drop-casting on the SPE/Au surface; in essence, this figure clearly points to the absence of agglomeration on the electrode surface, since CTAB promotes a better distribution of AuNPs.

EDX analysis was used to examine the chemical composition of the SPE/AuNPs/g-C_3_N_4_@CTAB surface. The results obtained are presented in Fig. 1D and I, Table S2 and Fig. S4, where the modifying elements are clearly visible; this essentially confirms that the modifications have occurred successfully, corroborating with the SEM images. Next, Raman spectroscopy was carried out in order to analyze the chemical structure of the sensing platform (Fig. 1J). The spectrum shows a typical graphite flake profile, with D and G bands at ~ 1330 cm^− 1^ and ~ 1580 cm^− 1^ and 2D band at ~ 2680 cm^− 1^, where the D band is attributed to sp³/sp carbon networks, edge defects, and heteroatom incorporation. The G band corresponds to sp²-hybridized carbon, while the 2D band corresponds to the overtone of the D and G bands, characteristic of graphitic structures [50–52]. The ratio of the intensity of the D-band to G-band (I_D_/I_G_) indicates that SPE/Au has less defects, with 0.11 I_D_/I_G_ ratio [53], while the SPE/AuNPs/g-C_3_N_4_ exhibits an I_D_/I_G_ ratio of 0.76, which points to an increase in the number of defects on the structure, possibly due to the aggregated nanosheets. Compared to only the g-C_3_N_4_, the SPE/AuNPs/g-C_3_N_4_ shows a slight decrease in I_D_/I_G_ ratio, which can be attributed to the electrostatic and supramolecular interactions promoted by CTAB promoting greater structural stabilization (decrease in ID band) and planarization effect with sheet stacking and increase in π-conjugation (increase in IG band) [29, 54]).

Lastly, contact angle measurements were carried out in order to evaluate the electrode wettability using ultra-pure water droplets, where 0 to 90º contact angles are considered hydrophilic and 90 to 180º are considered hydrophobic. The results obtained are shown in Fig. 1 K to M, where SPE/AuNPs exhibits a contact angle of 43.27º (Fig. 1K). This low contact angle is a result of higher surface energy and defects of the AuNPs clusters, which favor electrode surface wettability [55]. Upon introducing g-C_3_N_4_ into the SPE/AuNPs, the contact angle decreases to 38.18º (Fig. 1L); this result can be explained by the g-C_3_N_4_ structure, which is full of nitrogen sites that facilitate interaction with water molecules through hydrogen bonds, leading to enhanced wettability [56].

The contact angle of the SPE/AuNPs/g-C_3_N_4_@CTAB was found to have increased to 80.10º (Fig. 1M). Even though this value remains within the hydrophilic region, a considerable increase occurs when CTAB is added; this outcome points to the successful interaction of CTAB with the already modified electrode surface. As mentioned earlier, surfactants possess a CMC, where the micelles formation occurs (0.9 to 1 mmol L^− 1^ for CTAB), which makes the hydrophilic head point outward toward the solvent and the hydrophobic head point inward toward the center of the micelle. Thus, the increase in hydrophobicity indicates that no micelles were formed, despite the higher concentration of CTAB used compared to the data reported in the literature. However, the CMC value varies depending on thermodynamic factors such as temperature, pH, and electrolyte. Additionally, the polar characteristic of g-C_3_N_4_ favors interaction with the CTAB polar cationic head [57]. By virtue of that, CTAB monomers are assumed to interact with the electrode surface, forming a monolayer [42]. Although a more hydrophobic surface would, per se, hinder the interaction between an aqueous media and the electrode surface, as it is the case for the UA detection, the electrochemical response obtained from the addition of CTAB supports the decision of maintaining this configuration of modifiers, as it clearly enhances UA i_*p*_ response, facilitating the interaction of the analyte on the electrode surface.

### Electrochemical characterization of the SPE/AuNPs/g-C_3_N_4_@CTAB

**Fig. 2:**
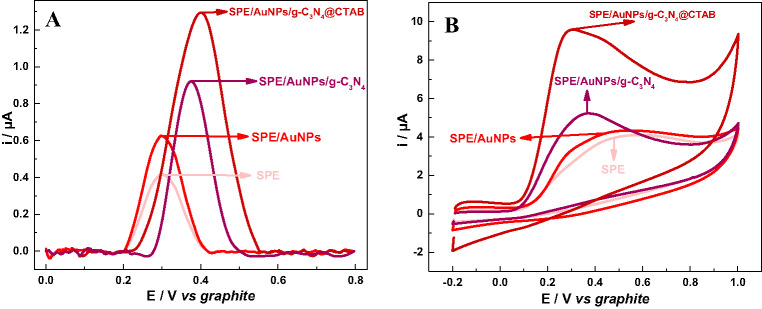
**A**) DPV and **B**) CV profiles for 200 µmol L^− 1^ UA in 0.1 mol L^− 1^ PBS at pH 7.0 in the study of the comparison of modifiers

The influence of the modifiers on the electrochemical performance of the SPE/AuNPs/g-C_3_N_4_@CTAB sensor applied for UA detection was analyzed by DPV and CV measurements using UA concentrations of 200 µmol L^−1^ and 1.0 mmol L^−1^, respectively, in 0.1 mol L^−1^ PBS at pH 7.0. In both techniques, when the unmodified SPE is compared to the SPE/AuNPs, an increase is observed in UA oxidation peak current (i_*p*_), as shown in Fig. 2; this increase in *i*_*p*_ is attributed to the increase in electrode surface area and conductivity, which facilitate electron transfer [15–17].

Upon the modification of SPE/AuNPs with g-C_3_N_4_, another increase is observed in UA i_*p*_, indicating the efficacy of the modifiers; this increase can be attributed to an increase in electroactive area caused by the incorporation of g-C_3_N_4_ into the electrode, which facilitated interaction with UA on the electrode surface and boosted charge transfer [58]. Furthermore, the nitrogen-rich framework of g-C_3_N_4_, composed of triazine and heptazine moieties possessing lone-pair electrons, promotes a strong affinity towards UA. This interaction is primarily driven by extensive hydrogen bonding between the nitrogen atoms of g-C_3_N_4_ and the amine/carbonyl functional groups of UA, alongside π - π stacking interactions between their respective planar conjugated ring systems. This strong structural compatibility enhances the accumulation of UA at the available adsorption sites on the electrode surface, thereby improving the oxidation kinetics and increasing the resulting i_p_ values [59].

The SPE/AuNPs/g-C_3_N_4_@CTAB exhibited a greater increase in UA oxidation i_*p*_ values, as can be seen from the CV and DPV profiles; this shows that the addition of CTAB jointly with g-C_3_N_4_ leads to better electrochemical performance. This fact can be attributed to the previously mentioned properties of CTAB, which forms a positively charged monolayer adhering to the g-C_3_N_4_ through its hydrophilic cationic head via electrostatic interactions, leading to a more stable, organized and distributed layer that helps the interaction between UA and g-C_3_N_4_, while increasing surface area, in addition to facilitating electron transfer [30, 31].

The observed potential shifts between DPV and CV arise because the E*p* of an irreversible process in CV is dictated by the scan rate and slow charge-transfer kinetics (related to molecular reorganization) [60, 61], whereas in DPV technique is governed by pulse amplitude and different measurement time scales [62]. The shift towards lower oxidation potentials at the SPE/AuNPs/g-C_3_N_4_@CTAB compared to the unmodified SPE implies that the nanostructured platform effectively facilitates faster electron transfer for UA oxidation despite the irreversibility of the reaction.

CV measurements were performed using 0.1 mol L^−1^ KCl as supporting electrolyte, in the presence of 1.0 mmol L^−1^ [Fe(CN)_6_]^3-^/[Fe(CN)_6_]^4-^, with scan rates ranging from 5 to 150 mV s^−1^, as shown in Fig. S5. A comparative analysis of the electrode configurations shows that SPE/AuNPs/g-C_3_N_4_@CTAB exhibited better electrochemical performance, which corroborates with what has been discussed previously. Furthermore, the electroactive area was calculated through the Randles-Sevick equation (Eq. 2) using the same redox probe [63].

2 $${\mathrm{i}}_p=\left(2.69\times10^5\right)\mathrm{n}^\frac32\mathrm{AD}^\frac12\mathrm{Cv}^\frac12$$

where i_*p*_ is anodic peak current (A), *n* represents the number of transferred electrons, *A* is the electroactive area (cm^2^), *D* is the diffusion coefficient of the redox pair (7.6 × 10^− 6^ cm^2^ s^−1^), *C* is redox pair concentration (mol cm^−3^), and v is the scan rate applied (V s^−1^). The estimated electroactive area for the SPE/AuNPs, SPE/AuNPs/g-C_3_N_4_, and SPE/AuNPs/g-C_3_N_4_@CTAB sensors were 0.58 cm², 0.63 cm², and 0.92 cm², respectively. These values represent an increase of 274.2%, 295.9%, and 428.4%, respectively, in the electroactive area of the modified SPEs compared to the unmodified SPE (0.19 cm²) [32].

**Fig. 3:**
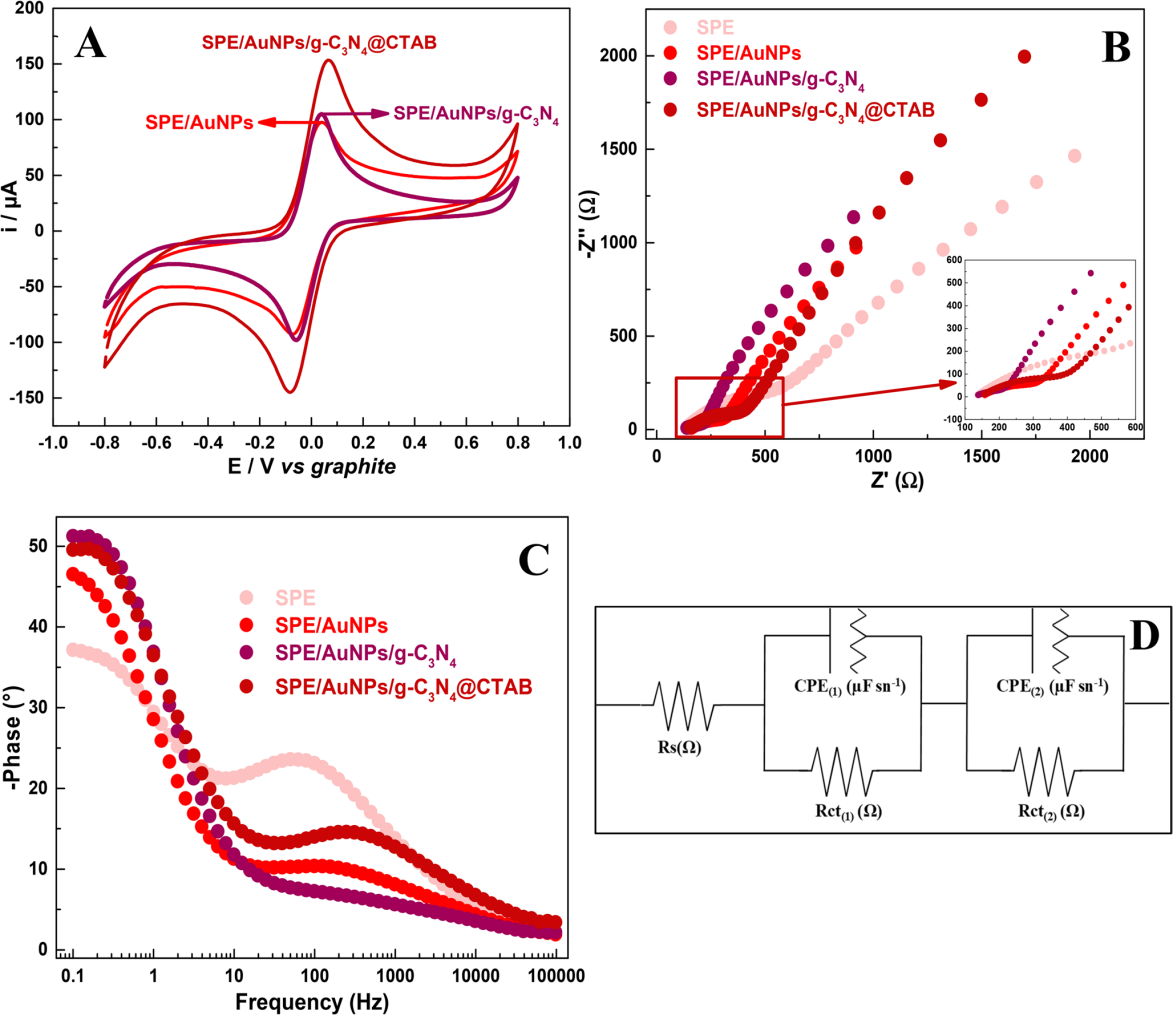
**A**) CV profiles comparing the i_*p*_ response of each configuration in Fe(CN)_6_]^3−^/[Fe(CN)_6_]^4−^on 0.1 mol L^−1^ KCl. **B**) Nyquist and **C**) Bode plots for different electrode configurations; **D**) the equivalent circuit

To assess the electrochemical behavior of each modification step, EIS analysis was carried out. Figure 3B and C show the Nyquist and Bode plots, respectively. The circuit equivalent system was subjected to a simulation analysis, based on the experimental data, where the proposed circuit is demonstrated in Fig. 3D and the calculated values are presented in Table 1.

**Table 1:** Circuit element proposal and their values from simulation of the EIS measurements

Circuit element	Electrode			
	Bare SPE	SPE/AuNPs	SPE/AuNPs/g-C_3_*N*_4_	SPE/AuNPs/g-C_3_*N*_4_@CTAB
R_s_ (Ω)	135.3	155.2	135.2	151.8
CPE_(1)_ (µF s^*n*−1^)	1.4 × 10^− 4^	1.9 × 10^− 4^	2.8 × 10^− 5^	8.9 × 10^− 5^
n_dl(1)_	0.57	0.50	0.45	0.53
R_ct(1)_ (Ω)	489.1	176.9	96	273.4
CPE_(2)_ (µF s^*n*−1^)	5.6 × 10^− 4^	1.0 × 10^− 3^	9.5 × 10^− 4^	5.2 × 10^− 4^
n_dl(2)_	0.68	0.74	0.79	0.73
R_ct(2)_ (Ω)	6940	7256	5501	14,968
χ²	1.6 × 10^− 4^	4.6 × 10^− 4^	1.9 × 10^− 4^	2.4 × 10^− 4^

The same circuit system was applied in the simulation for all electrodes, for comparison purposes. The circuit model described represents a modified electrode surface with two parallel resistance-constant phase element (R-CPE) branches connected in series. The first, high-frequency branch (Rct_(1)_​//CPE_(1)​_), models fast processes like charge transfer and the immediate double-layer response on the electrode surface. The second, low-frequency branch (Rct_(2)_​//CPE_(2)_​), accounts for slower phenomena, such as diffusion, adsorption, or ion movement within a porous film. This system of two parallel R-CPE circuits is described in the literature as a simultaneous process that occurs on the electrode surface and is separated by the time constant effect [64]. 

For the bare electrode, both resistive contribuitions were high Rct_(1)_= 489.1 Ω and Rct_(2)_ = 6,940 Ω); this outcome points to slow electron transfer on the electrode surface and poor capacitive response, in line with the low voltammetric currents. After AuNPs electrodeposition, Rct_(1)_ dropped substantially to 176.9 Ω, highlighting the catalytic role of AuNPs in accelerating fast charge-transfer pathways. Meanwhile, Rct_(2)_ remained high (7256 Ω); this shows that deeper interfacial processes, likely linked to the varnish-graphite matrix, were not fully improved by the electrode modification with Au. The increase in CPE_(1)_ and CPE_(2)_ confirmed that AuNPs expanded the electroactive surface area and improved charge accumulation, consistent with the Bode plot that exhibited enhanced phase angles at intermediate frequencies.

The addition of g-C₃N₄ further lowered Rct_(1)_ to 96 Ω, the smallest among all the electrodes; this is indicative of an efficient fast electron transfer mediated by the nitrogen-rich nanosheets. At the same time, Rct_(2)_ also decreased (5501 Ω); this implies that g-C₃N₄ contributed not only to surface catalysis but also to improved ion redistribution at slower time scales. The higher ndl_(2)_ value (0.79) pointed to a more ideal capacitive behavior in this low-frequency regime, reflecting enhanced adsorption of ionic species and favorable surface chemistry. This dual improvement corroborates the CV and DPV results, where g-C₃N₄ promoted UA adsorption and facilitated oxidation. However, the reduction in CPE_(1)_ values suggested that nanosheet aggregation partially limited accessible double-layer regions, observed in the SEM images of clustered g-C₃N₄ domains.

In contrast, the SPE/AuNPs/g-C₃N₄@CTAB electrode exhibited a higher Rct_(1)_ (273.4 Ω) compared to g-C₃N₄ alone, which may be attributed to the formation of a CTAB monolayer that introduced additional resistance at the electrode/electrolyte boundary. However, CPE_(1)_ increased substantially (8.9 × 10⁻⁵ µF sⁿ⁻¹), evidencing improved wettability and double-layer stability. Interestingly, Rct_(2)_ rose sharply to 14968 Ω; this suggests that CTAB altered the slow interfacial dynamics, possibly through the creation of organized surfactant domains or partial micelle-like structures that impede diffusion at longer timescales. The same behavior has been described by Razzaq et al. (2024), where the authors investigated the electrochemical performance of CTAB on WO_3_ films [65]. It should be noted, however, that the Bode plot revealed a broad and stable phase response; this clearly shows that the surfactant effectively homogenized the g-C₃N₄ layer, improving its capacitive performance. This behavior demonstrates that UA detection at this modified surface is not strictly governed by the baseline electron-transfer resistance. While the CTAB monolayer introduces an interfacial barrier that increases Rct, its cationic nature significantly enhances the surface wettability and promotes strong electrostatic attraction towards UA. Consequently, the substantial increase in i_*p*_ observed in the CV and DPV measurements is an adsorption-driven enhancement. The effective adsorption and interaction of UA at the available active sites of the AuNPs/g-C₃N₄@CTAB surface thoroughly compensates for the higher interfacial resistance, indicating that specific surface affinity and optimized analyte adsorption results in a higher electrochemical performance towards UA oxidation.

### Effect of pH and scan rate on UA electrochemical detection

**Fig. 4:**
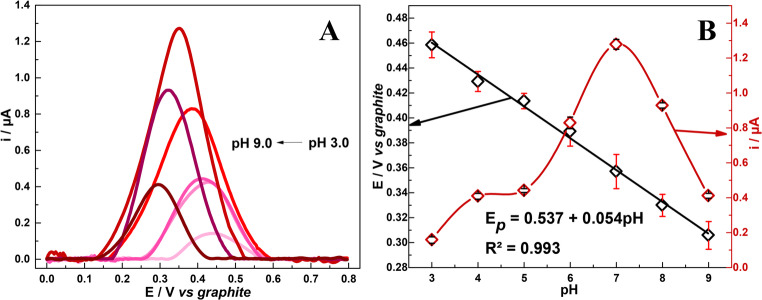
**A**) DPV profiles for 200 µmol L^−1^ UA in 0.1 mol L^−1 ^PBS varying pH value from 3 to 9 and **B**) relation between pH, E_*p*_ and i_*p*_ values

pH is a major factor in electrochemical sensing, since it dictates the concentration of protons and electrons in the solution, which affects the electrochemical behavior of the electrode. Taking this into account, DPV measurements were performed in order to determine the optimum pH value for the degradation of 200 µmol L^−1^ UA in 0.1 mol L^−1^ PBS. The DPV analysis was carried out by testing the pH range of 3.0 to 9.0, and the results obtained are shown in Fig. 4A and B. It is clear that pH 7 is the optimal pH, since the application of this pH level resulted in the highest oxidation i_*p*_ response for UA, while more acidic and basic pH levels led to a considerable decrease in i_*p*_ value.

These results can be explained by the fact that UA is a weak diprotic acid, which is characterized by two dissociations, with two pKa values: ~5.4 and ~ 9.8. Thus, at pH 7.0, UA is found as a negatively charged deprotonated urate anion, its most electroactive form; in this condition, the compound promotes electrostatic attraction with the positively charged layer of CTAB, facilitating charge transfer and enhancing oxidation (i_*p*_*)*. While at pH below 5.0 (first pKa), UA exists mostly in its fully protonated form, with lower solubility, which results in a slower charge transfer and electrostatic repulsion. At pH 9.0, UA is closer to its second deprotonation (pKa 9.8), being less electroactive; and since UA is a diprotic weak acid, its oxidation process involves two protons and two electrons. It is worth noting that, at pH 9.0, proton availability is lower, and this influences the oxidation process while decreasing the current response [14, 66–68].

**Scheme 1:**
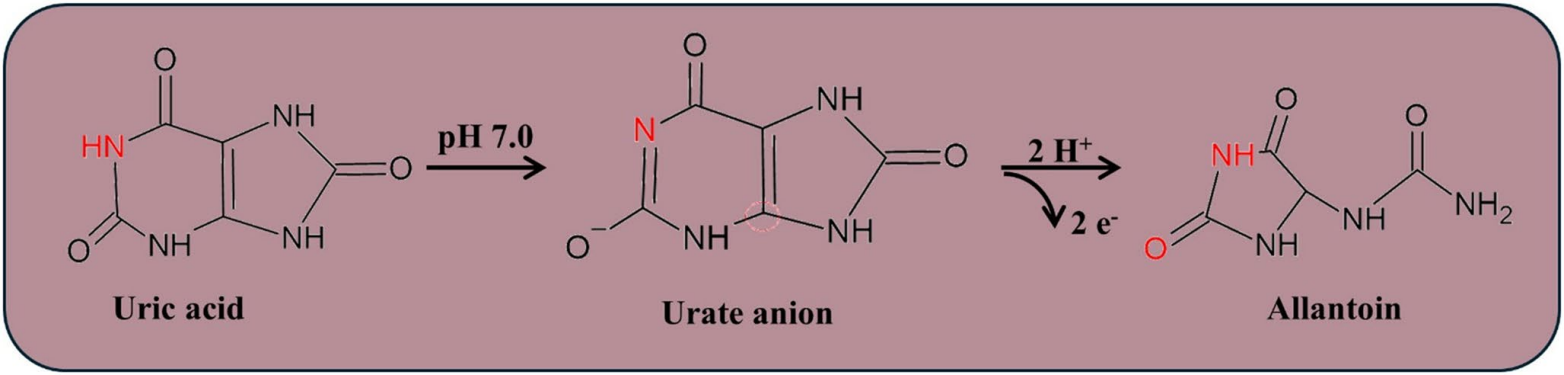
Electrochemical oxidation mechanism of UA on SPE/AuNPs/g-C_3_N_4_@CTAB

Given that the electrochemical oxidation of UA involves two protons and two electrons, at pH 7.0, where UA is found in the form of urate anion, the acid is oxidized to allantoin (see Scheme 1) [69]. This can be further confirmed when one looks at the linear relation between pH and E*p*, where E*p* shifts to less positive values as pH increases, with a slope value of 0.054 V pH^− 1^ (R² = 0.993), which is close to the theoretical value of 0.059 V pH^− 1^, based on the Nernst equation.

**Fig. 5:**
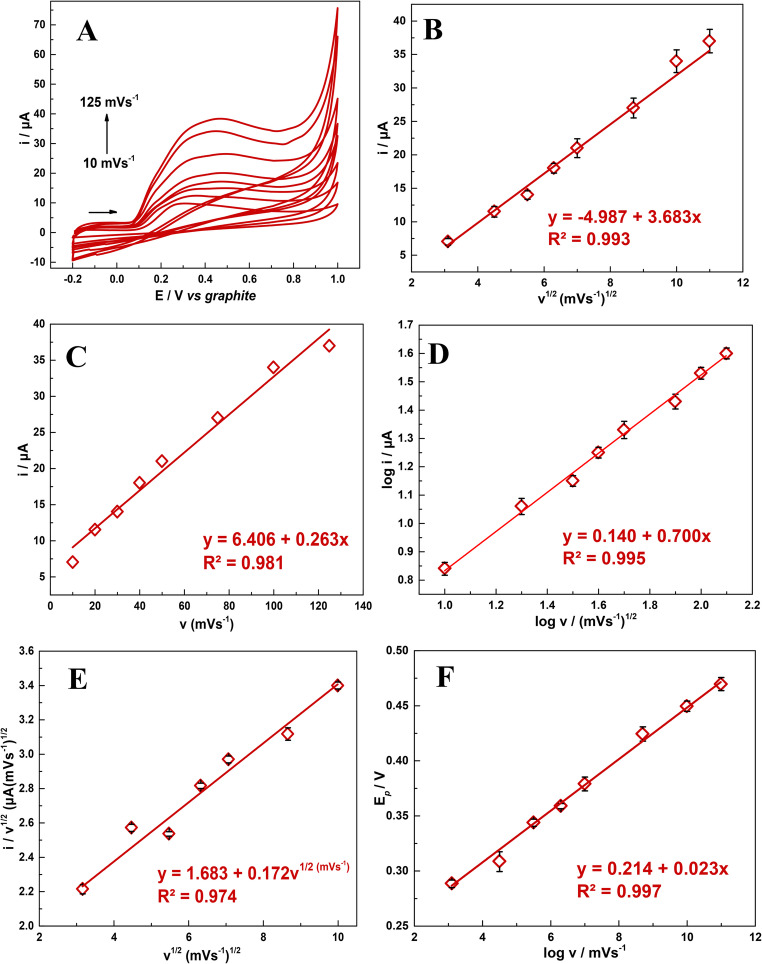
**A**) CV profiles from 10 to 125 mVs^−1^ for 1.0 mmol L^−1^ UA in 0.1 mol L^−1^ PBS at pH 7.0; linear relation between**B**) i_*p*_ and *v*^1/2^; **C**) i_*p*_ and *v*; **D**) log i_*p*_and log *v* and **E**) i_*p*_/*v*^1/2^ and *v*^1/2^ and **F**) E_*p*_ and log *v*

To analyze the mechanism involving UA oxidation on the surface of the SPE/AuNPs/g-C_3_N_4_@CTAB, CV analyses were performed using 0.1 mol L^− 1^ PBS, at pH 7.0, in the presence of 1.0 mmol L^− 1^ UA, with scan rates ranging from 10 to 125 mV s^− 1^. The results obtained are presented in Fig. 5A to F. Figure 5F shows that an increase in scan rate leads to an increase in UA i_*p*_ values, and Fig. 5B to C clearly points to a linear relation between square root of scan rate and UA i_*p*_, as well as a linear relation between scan rate and UA i_*p*_ with the coefficient of determination (R²) = 0.993 and 0.981, respectively. Furthermore, as can be seen from log i_*p*_ *versus* log *v* (Fig. 5D) there is a linear relation between the two variables, expressed by the equation of *log* i_p_/µA (UA) = 0.170 + 0.700 log *v*/mV s^− 1^, with excellent linearity (R^2^= 0.997); considering that the slope (0.700) value lies between 0.50 and 1.0 − theoretical values in Laviron’s theory [70], the oxidation process points towards a mixed-controlled mechanism.

To estimate the proportion of adsorption and diffusion contribution to i_*p*_ values, the results obtained in Fig. 5E shows a linear relation between i_*p*_/*v*^1/2^ and *v*^1/2^ (R² = 0.974), expressed in Eq. 3, varying from 10 to 100 mVs^− 1^ due to the fact that in 125 mV s^− 1^ there is a influence of uncompensated resistance and inherent kinetics limitations. With these data obtained, the application of the equation proposed by Dunn et al. (Eq. 4) [71] and its derivation (Eq. 5).

3 $${\mathrm{i}}_p/v^\frac12=1.683+0.172v^\frac12$$

4 $${\mathrm{i}}_p/v^\frac12={\mathrm{k}}_{1v}^\frac12+{\mathrm{k}}_2$$

where:

- i is current response (mVs^−1^);
- *v*^1/2^ is the square root of scan rate;
- k_1_*v*^1/2^ is the constant corresponding to the adsorption process;
- k_2_ is the constant corresponding to the diffusion process.

5 $${\mathrm{i}}_{total}={\mathrm{i}}_{adsorption}+{\mathrm{i}}_{diffusion}$$

where:

- i_adsorption_ = Eq. 3 slope value × scan rate (50 mVs^−1^).
- i_diffusion_ = Eq. 3 intercept value × the square root of scan rate (50 mVs^−1^)

At a scan rate of 50 mV, the equation reveals that the surface-controlled process accounts for 41.95% of the total current, while the diffusion-controlled process accounts for 58.95%. This proportion corroborates with the results obtained in Fig. 5B C, as well as Fig. 5D, indicating a mixed-controlled mechanism for the oxidation of UA.

Figure 5 F shows the relation between E_*p*_ and log *v*, expressed by the equation of *log E*_*p*_ (UA) = 0.214 + 0.023 log *v*/mV s^−1^, with good linearity (R^2^= 0.997); where UA oxidation potential shifts towards more positive values, as log *v* increases; in essence, these results are indicative of the influence of scan rate in the transport mechanism of UA oxidation process, especially on carbon electrodes, where higher scan rates can change this mechanism.

To confirm the number of electrons (*ne*^-^) involved in the UA oxidation process, Eq. 6 was applied. Considering that the redox process of UA is irreversible, the charge transfer coefficient (α) was estimated to be 0.5 [72], where E_*p*_ and E_*p/2*_ were + 0.284 V and + 0.243 V (*versus* graphite), respectively. This analysis yielded an *n* value of 2.1, which is quite close to 2.0.

6 $$\:{E}_{p\:-\:}{E}_{p1/2}=\frac{47.7}{(\alpha\:\:\times\:n)}\mathrm{m}\mathrm{V}$$

### SPE/AuNPs/g-C_3_N_4_@CTAB electroanalytical performance and UA determination in human blood, serum and plasma samples

**Fig. 6:**
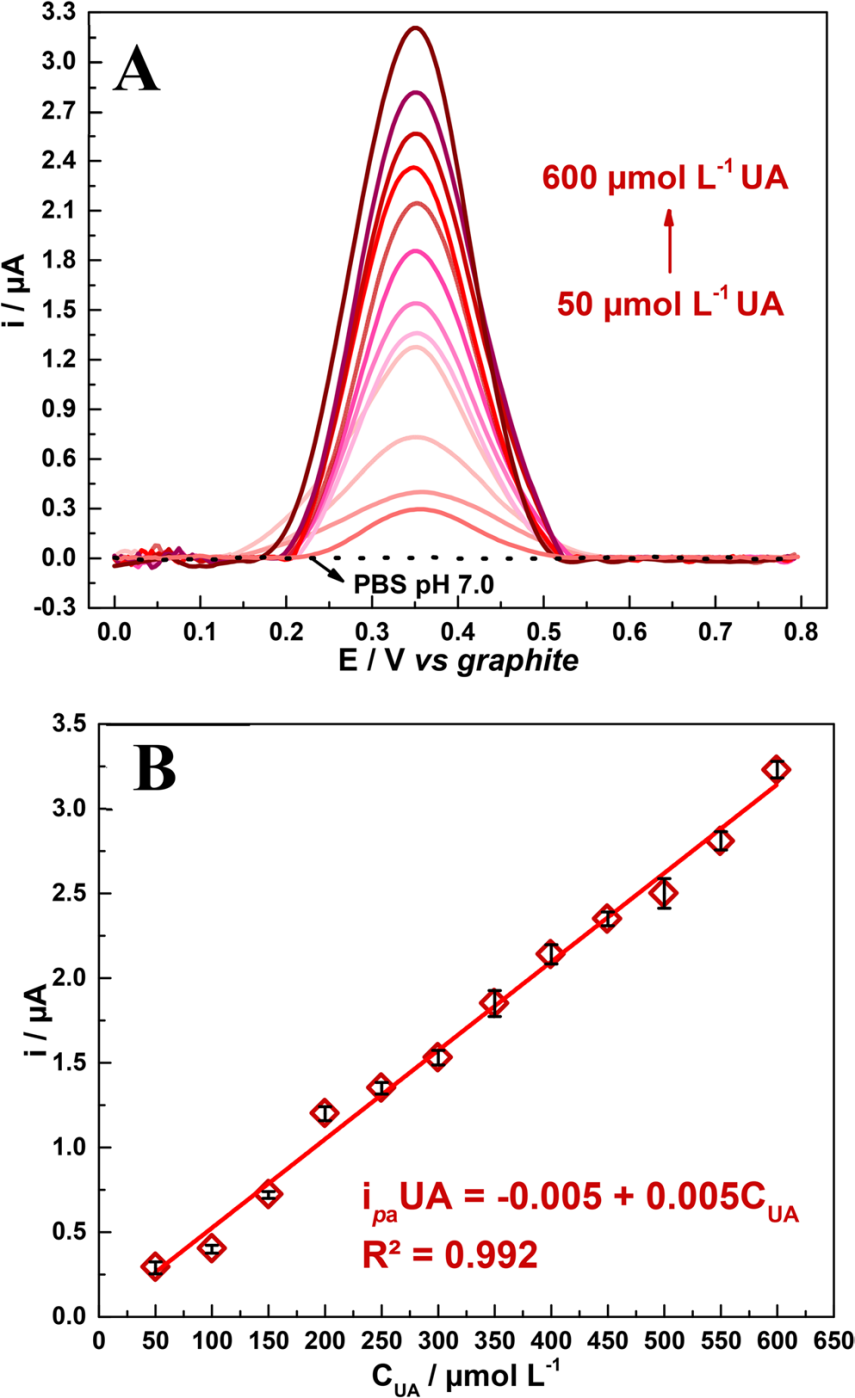
**A**) DPV profiles for different concentrations of UA in 0.1 mol L^− 1^ PBS at pH 7.0 and **B**) calibration curve for i_*p*_ and UA concentration (C_UA_/µmol L^− 1^)

Through the application of DPV, an analytical curve was constructed for UA concentration within the range of 50 to 600 µmol L^−1^ using 0.1 mol L^−1^ PBS at pH 7.0 as the supporting electrolyte – see the results in Fig. 6. This specific concentration range was tested because the reference UA concentration levels in human blood lies within ~200 to 500 µmol L^−1^, as previously mentioned, the test was conducted focusing primarily on the sensing device applicability. Fig. 6A shows the DPV profiles obtained from the analysis conducted; one will observe that, as UA concentration increases, there is a linear increase between the current response and the applied potential, which is confirmed by Fig. 6B. The fitted equation was expressed as i_*p*_/µA (UA) = –0.0054 ± 0.0536 + 0.0052 ± 0.0001 C_UA_/µmol L^−1^; this equation exhibits a good linear correlation between i_*p*_ and µA (R² = 0.992).

The LOD obtained from the application of Eq. 7 was 1.95 µmol L^− 1^; and the limit of quantification (LOQ), calculated by Eq. 8, was 6.50 µmol L^–1^. Additionally, sensitivity (*S*) was calculated by Eq. 9, and its value was found to be 0.005 µA µmol L^− 1^ cm^− 2^. The LOD, LOQ and *S* values shows that the modified SPE was sensitive to different concentrations within the proposed range.

7 $$LOD=3S_b/b$$

8 $$\mathrm{LOQ}=10S_b/b$$

9 $$S=b/A$$

where S_*b*_ is the standard deviation of 10 measurements of blank sample (0.1 mol L^−1^ PBS at pH 7) and *b* is the slope of the analytical curve, and *A* is the electrode electroactive surface area.

Different analytical and electroanalytical platforms have been proposed in recent years for monitoring uric acid in different samples, as shown in Table 2. Devices based on commercial platforms such as glassy carbon electrodes (GCE) and low-cost platforms such as SPE have been used in approaches with different electrode surface chemical modifiers. Beyond electroanalytical strategies, conventional chromatographic methodologies, such high-performance liquid chromatography (HPLC), remain established standards for UA determination. The data outlined in the table indicate that the proposed electrochemical device demonstrates comparable analytical capabilities to these conventional techniques. Overall, the SPE/AuNPs/g-C_3_N_4_@CTAB sensor has a dynamic working range comparable to the majority of reported devices, which has allowed its simple and efficient application to blood samples. Conversely, some reported sensors have considerably lower ranges, requiring successive sample dilution steps. Additionally, the proposed sensor showed excellent detectability, with LOD in the same order of magnitude as (bio)sensors modified with similar nanomaterials such as AuNPs, g-C_3_N_4_ and CTAB. Importantly, the device proposed in this study offers a relatively simple construction process and can be directly applied to analytical determinations after a simple dilution step in supporting electrolyte. Therefore, we strongly believe that the SPE/AuNPs/g-C_3_N_4_@CTAB sensor is a promising analytical tool for (bio)analysis with suitable characteristics such as precision and accuracy for monitoring uric acid in complex blood samples.

**Table 2:** Comparison of the electrochemical performance of SPE/AuNPs/g-C_3_N_4_@CTAB with other devices reported in the literature for UA monitoring in biological samples

Technique	LOD (µmol L^− 1^)	Linear range (µmol L^− 1^)	Samples	Reference
Qr-Fe_3_O_4_/SPCE	0.08	0.2–25	Spiked urine	[73]
Unmodified DS-SPCE	0.10	0.5–41.5	Human urine	[74]
HG/StPE	0.15	2–10	Synthetic urine	[75]
CTCPE	1.70	5–125	Herring sperm DNA and human blood serum	[76]
CZTO/MWCNT/CPE	5.76	10–100	Urine	[77]
g-C_3_N_4_ NS/GCE	4.45	100–1000	Spiked urine	[78]
Pt@NP-AuSn/Ni/CFP	0.67	25–800	Urine	[79]
Uricase/CNT-CMC/Au	2.80	20–2700	Human serum and urine	[80]
SPE/Uricase/MWCNTs	0.33	5–1000	Human saliva	[81]
PtNPs/MWCNT/SPCE	0.49	5–690	Synthetic urine	[82]
OSC-PLS/GCE	3.10	10–210	Human plasma and urine	[83]
ZIF-L (Zn)/SPGE	0.04	0.1–900	Human urine	[84]
HILIC	0.59	0.0–118.97	Bovine milk and orange	[85]
HPLC-UV	0.31	10–500	Human plasma	[86]
HPLC	5.5	4–538	Microalgae	[87]
SPE/AuNPs/g-C_3_N_4_@CTAB	1.95	50–600	Human whole blood, serum, and plasma	This work

*ZIF-L (Zn)/SPGE* zeolitic imidazolate framework-L (Zn) modified screen-printed graphite electrode, *Qr-Fe*_*3*_*O*_*4*_*/SPCE* screen printed carbon electrode modified with quercetin functionalized-iron oxide nanoparticles, *Unmodified DS-SPCE* DropSens screen-printed carbon electrode, *HG/StPE* stencil-printed electrode with a hydrogel, *SPE/Uricase/MWCNTs* multi-wall carbon nanotube–modified screen-printed electrode immobilized by uricase, *PtNPs/MWCNT/SPCE* screen-printed carbon electrodes modified with multi-walled carbon nanotubes and platinum nanoparticles, *Pt@NP-AuSn/Ni/CFP* Pt nanoparticles modified nanoporous AuSn alloy on Ni buffered flexible carbon fiber paper, *CTCPE* cationic surfactant cetyltrimethylammonium bromide modified carbon paste electrode, *Uricase/CNT-CMC/Au* uricase/carboxymethylcellulose dispersed carbon nanotube/gold thin film biosensor, *OSC-PLS/GCE* glassy carbon electrode coupled with orthogonal signal correction-partial least squares, *g-C*_*3*_*N*_*4*_ *NS/GCE* graphitic-like carbon nitride nanosheets on glassy carbon electrode, *CZTO/MWCNT/CPE* carbon paste electrode modified with calcium zirconium titanium oxide multiwalled carbon nanotubes nanocomposites, *HILIC* hydrophilic interaction liquid chromatography, *HPLC-UV* High performance liquid chromatography-ultraviolet, *HPLC* high performance liquid chromatograph

**Fig. 7:**
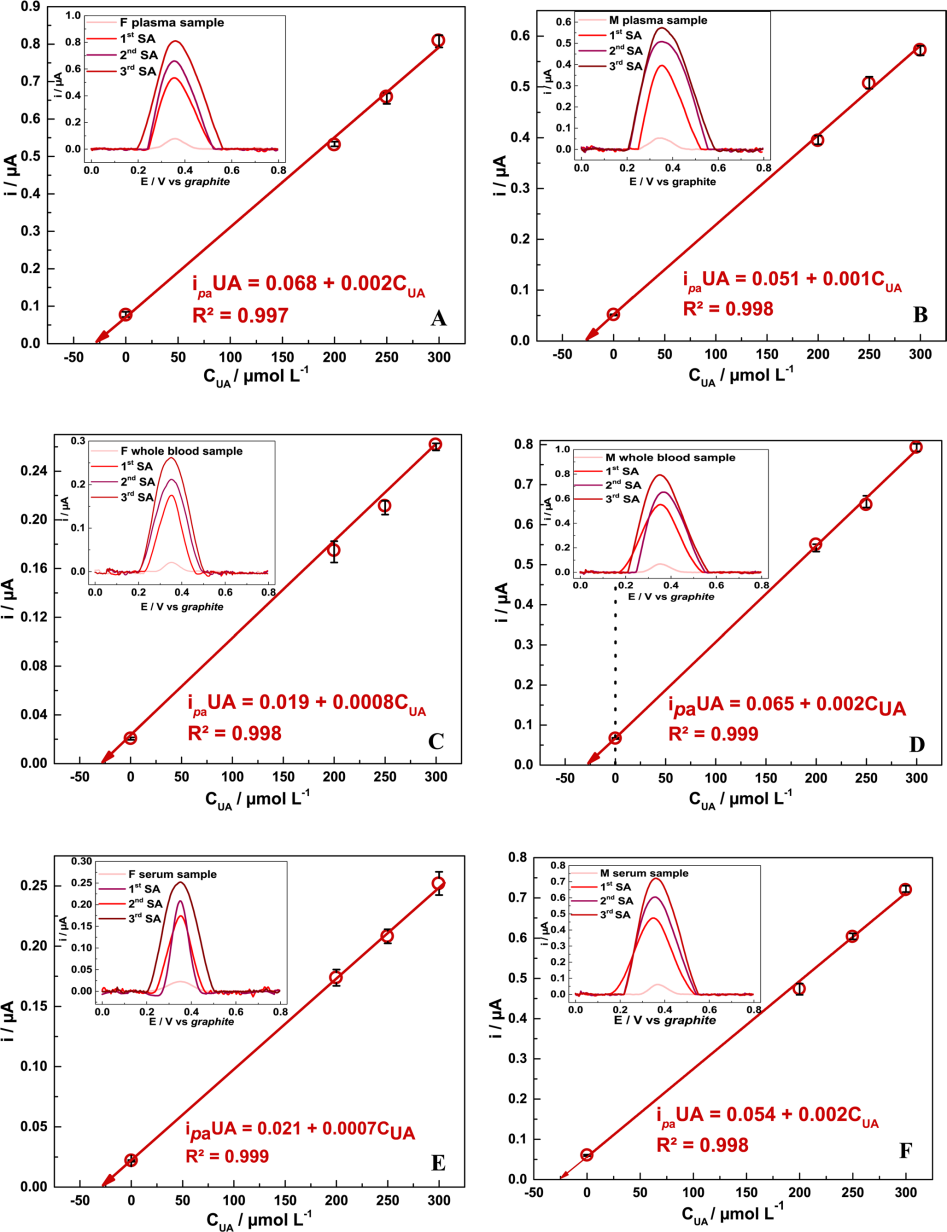
Linear relation between i_*p*_ response and UA concentration on real matrices. Plasma: **A**) female, **B**) male; Whole blood: **C**) female, **D**) male; Serum: **E**) female, **F**) male. Insets: represent the DPV profiles for each sample: 1^st^ standard addition (SA) (200 µmol L^− 1^ UA), 2^nd^ SA (250 µmol L^− 1^ UA) and 3^rd^ SA (300 µmol L^− 1^ UA)

UA determination was carried out by DPV analysis using the standard addition method and linear extrapolation, with 3 additions of 50 µmol L^− 1^ UA, starting from 200 to 300 µmol L^− 1^ in human female and male whole blood, serum, and plasma samples, as shown in Fig. 7. The results obtained from this analysis point to the high electrochemical efficiency of the SPE/AuNPs/g-C_3_N_4_@CTAB device when applied for the determination of the analyte in blood samples, as shown in Table 3. Additionally, the samples were also analyzed using enzymatic colorimetric assays.

**Table 3:** Determination of UA on male and female human whole blood, serum and plasma samples by DPV on SPE/AuNPs/g-C_3_N_4_@CTAB

Samples	Proposed method				
	Found concentration (µmol L^− 1^)	Found Concentration (mg dL^− 1^)	Comparative method (mg dL^− 1^)	SD (mg dL^− 1^)	t_calc_
Female plasma	287.93	4.84	N/A	± 0.51	-
Male plasma	267.89	4.53	N/A	± 0.08	-
Female whole blood	285.20	4.79	N/A	± 0.41	-
Male whole blood	272.44	4.62	N/A	± 0.11	-
Female serum	274.25	4.61	4.80^*^	± 0.28	1.77
Male serum	248.60	4.18	4.20^**^	± 0.03	0.95

t_crit_ (95%) = 4.3; ^*^285.3 µmol L^− 1^ UA,^**^249.83 µmol L^− 1^ UA

The results obtained from both techniques were then statistically compared. The student’s *t*-test at 95% confidence level exhibited a calculated *t*-value of 1.77 and 0.95 for female and male blood donors, whereas the critical *t*-value was 4.30. Thus, based on these results, one can conclude that there was no significant difference between the two techniques evaluated; this suggests that the proposed method provides excellent accuracy and can be applied for predictive electrochemical analyses. Although UA concentration levels found in both tests were higher for the female subject (4.8 mg dL^− 1^) than the male subject (4.2 mg dL^− 1^), both were found to be within the known UA range of – 2.0 to 6.0 and 2.0 to 7.0 mg dL^− 1^, respectively [88–91]. Even though the samples analyzed in this study are complex matrices, the SPE/AuNPs/g-C_3_N_4_@CTAB exhibited good sensitivity towards UA, equivalent to the method widely used for UA commercial clinical monitoring.

### Analyzing the stability, reproducibility and selectivity of the SPE/AuNPs/g-C_3_N_4_@CTAB

The electrochemical stability of the SPE/AuNPs/g-C₃N₄@CTAB sensor was analyzed by investigating its memory effect, specifically the degree of signal carry over from preceding measurements conducted at different concentrations, as significant inaccuracies in UA measurements may result in misdiagnosis and improper patient management. DPV measurements were performed using the same electrode with increasing and decreasing UA concentrations in the following order: 1st: 200 µmol L^− 1^; 2nd: 250 µmol L^− 1^; 3rd: 300 µmol L^− 1^; 4th: 350 µmol L^− 1^; and 5th: 200 µmol L^− 1^.

**Fig. 8:**
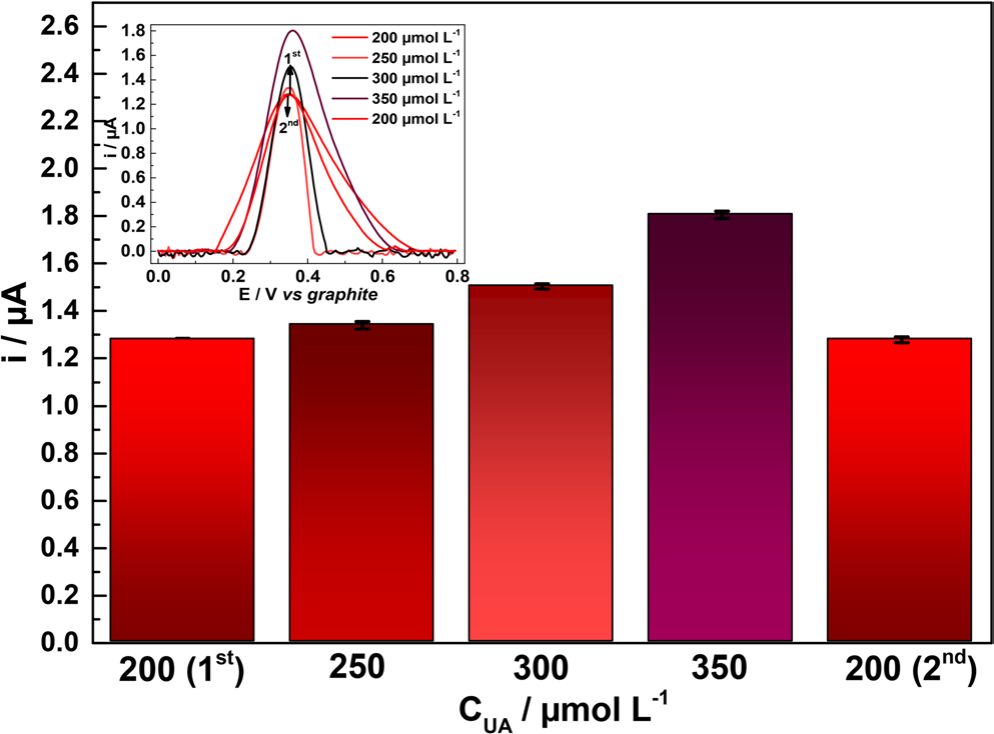
Memory effect study in four consecutive upscaling concentrations (200 to 350 µmol L^− 1^ UA)

As shown in Fig. 8, a comparative analysis of the first and fifth measurements revealed no significant variation in the UA i_*p*_ values; essentially, this points to the stability of the sensor when applied in consecutive measurements with varying UA concentrations. Additionally, to elucidate the platform reproducibility, an analysis using five different SPE/AuNPs/g-C_3_N_4_@CTAB was carried out by DPV for 200 µmol L^− 1^ UA in 0.1 mol L^− 1^ PBS pH 7.0. The results obtained are shown in Fig. S6, and the platform presents to be reproductible across five electrodes, with minimal disturbance on i_*p*_ response (RSD = 2.84%).

Lastly, to evaluate the selectivity of the sensor, a study was conducted using compounds commonly present in human blood, serum, and plasma as potential interferents, in a concentration ratio of 1:10 (50 µmol L^− 1^ UA: interferent) which encompasses or exceeds the physiological concentration of the majority of the interferents used [92]. As shown in Table 4, the sensor demonstrated high selectivity for UA, with minimal interference and low error values (–4.41 to 4.52%).

**Table 4:** UA electrochemical response in the presence of interferents in a 1:10 (UA: interferent) proportion

Interferent	i (µA) error (%) *
Glucose	−1.63
Urea	−0.35
Dopamine	+ 4.52
Progesterone	+ 1.51
Estriol	+ 1.59
Na^+^	−4.41
K^+^	+ 1.40
Ca^+^	−0.27
Mg^2+^	+ 1.85
Folic Acid	+ 0.95
Ascorbic Acid	+ 0.33
Creatinine	+ 0.09

*Error= [(Analytical signal presence – Analytical signal absence) ÷ Analytical signal absence) × 100%

It is worth noting that metallic cations, with the exception of Ca^2+^, recorded higher error values; this outcome can be attributed to the fact that these metallic species can be easily adsorbed on the electrode surface, blocking active sites and competing with UA, which is the case of Na^2+^. In this case, where there is a blockade of UA interaction with the electrode surface, the UA *i*_*p.a.*_ decreases [93]; however, where there is an increase in UA *i*_*p.a.*_, as in cases involving Mg^2+^ and K^+^, the metallic cations are found to be most stable in their fully charged form and do not take part in the redox process, but rather act to facilitate ion transport and UA interaction on the electrode surface [94, 95]. As for dopamine, the increase in its UA *i*_*p.a.*_ can be linked to the fact that the oxidation process exhibits a synergistic effect on UA oxidation on the electrode surface, which results in an increase in current response [96]. While urea and glucose were present at lower than physiological concentrations, it is important to note that neither compound is electroactive in our proposed system. The use of these sub-physiological concentrations is well-supported by existing literature, which frequently uses them when evaluating urea and glucose as interferents in UA electrochemical sensors [97, 98, 99, 100]. As for the inorganic analytes Na, K, Ca and Mg, none showed significant interference in the tests conducted.

### Theorical insights into UA adsorption by DFT analysis

To complement our experimental findings and gain atomistic-level understanding of the enhanced electrochemical response, DFT calculations were performed to investigate the adsorption behavior of UA onto the sensor surface, both with and without the CTAB modifier. An initial test for UA adsorption on the pristine AuNPs surface yielded a calculated adsorption energy of −0.84 eV, confirming that AuNPs alone provides a weaker interaction compared to the composite systems.

The calculated *E*_*ads*_ for UA on the AuNPs/g-C_3_N_4_ model was − 1.36 eV, indicating a favorable and spontaneous adsorption process. However, with the introduction of CTAB, forming the Au/g-C_3_N_4_@CTAB system, the adsorption energy increased significantly to −1.59 eV. This stronger adsorption (−0.23 eV difference) provides a fundamental theoretical explanation for the superior electrochemical performance observed experimentally.

The enhanced adsorption can be primarily attributed to the modification of the electronic structure at the electrode interface by CTAB. The surfactant’s positively charged hydrophilic head group creates a localized cationic field, which electrostatically attracts the deprotonated urate anion (the predominant form of UA at physiological pH 7.0, as confirmed by the pH optimization studies). This electrostatic facilitation aligns perfectly with the experimental observations from DPV and EIS analysis, where the CTAB-modified sensor showed a marked increase in peak current and a more favorable charge-transfer environment.

Furthermore, the DFT results corroborate the morphological characterization. The SEM images and contact angle measurements indicated that CTAB promotes a more homogeneous distribution of the g-C_3_N_4_ nanosheets and alters the surface wettability. Theoretically, this organized monolayer mitigates the agglomeration of g-C_3_N_4_, thereby exposing a greater number of active sites for UA interaction, as reflected in the higher (more negative) *E*_*ads*_. This synergy between the increased electroactive surface area (quantified experimentally as 0.92 cm^2^ for the full modified sensor) and the stronger adsorption energy underpins the sensor’s enhanced sensitivity.

The stronger binding in the presence of CTAB also rationalizes the excellent selectivity demonstrated in the interference study. The specific, energetically favorable interaction with UA, driven by both electrostatic complementarity and optimized surface geometry, makes the sensor less susceptible to signal disruption from common interferents like ascorbic acid, dopamine, and ions, which do not experience this specific, enhanced adsorption.

## Conclusion

The SPE/AuNPs/g-C_3_N_4_@CTAB electrochemical device was successfully developed and applied for the determination of UA in human blood samples. The incorporation of the modifiers into the SPE helped enhance the electroactive surface area and conductivity, in addition to facilitating interactions at the electrode/solution interface while also being cost-effective. Additionally, the developed sensor exhibited good sensitivity and detectability for UA, in the linear range of 50 to 600 µmol L^−1^, with an LOD and LOQ of 1.95 and 6.50 µmol L^−1^, which lied within the clinical reference values for UA in human blood, serum, and plasma samples, with a *S* of 0.005 µA µmol L^−1^ cm^−2^. The results obtained from the application of the SPE/AuNPs/g-C_3_N_4_@CTAB were validated via a commercial technique (enzymatic colorimetric), which indicated that the proposed method is comparable to the method currently applied in laboratorial contexts. Also, the SPE/AuNPs/g-C_3_N_4_@CTAB exhibited high stability and selectivity for UA. These results show that the modified SPE is a simple, quick, and cost-effective lab-made alternative sensing device that can be effectively applied for UA determination in clinical samples.

It is also important to mention that, the DFT calculations confirm that CTAB is not a passive component but an active modifier that strengthens the interaction between UA and the sensor surface. The more favorable adsorption energy of −1.59 eV for the AuNPs/g-C_3_N_4_@CTAB system directly correlates with the experimental enhancements in sensitivity, selectivity, and electron-transfer kinetics, providing a solid theoretical foundation for the superior analytical performance of the proposed electrochemical platform.

## Supplementary Information

Below is the link to the electronic supplementary material.

Supplementary Material 1

## Data Availability

No datasets were generated or analysed during the current study.
