# Supplementary material for: Development of a portable label-free electrochemical sensor modified with AuNPs/g-C3N4@CTAB for uric acid detection in complex blood samples
Source: Mikrochim Acta. 2026 Apr 24;193(5):344. doi: 10.1007/s00604-026-08027-1 (PMC13109136; doi:10.1007/s00604-026-08027-1)
Supplement: Supplementary file 1 — Supplementary Material 1 [file 604_2026_8027_MOESM1_ESM.docx]

**Development of a portable label-free electrochemical sensor modified with AuNPs/g-C_3_N_4_@CTAB for uric acid detection in complex blood samples**

Fernando C. Gallina^1^, Igor. G. S. Oliveira^1^, Muriel A. Rodrigues^1^, Luís F. Basso^1^, Oswaldo C. Junior^2^, Beatriz T. Marin^2^, Robson S. Souto^2^, Leandro M.C. Pinto^3^, Herintha C. Neitzke-Abreu^4^, Thalles P. Lisboa^1^, Marcos R. V. Lanza^2^, Willyam Róger Padilha Barros^1*^

^1^*Faculty of Exact Sciences and Technology, Federal University of Grande Dourados – UFGD, Rodovia Dourados-Itahum, km 12, Dourados, MS, 79804˗970, Brazil*

^2^*São Carlos Institute of Chemistry, University of São Paulo, Av. João Dagnone, 1100, São Carlos, SP, 13563˗120, Brazil*

^3^*Institute of Chemistry, Federal University of Mato Grosso do Sul, Av. Costa e Silva, s/nº, Campo Grande, MS, 79070-900, Brazil*

^4^*Faculty of Health Sciences, Federal University of Grande Dourados – UFGD, Rodovia Dourados-Itahum, km 12, Dourados, MS, 79804˗970, Brazil*

*Corresponding author:

E**˗**mail address: willyambarros@ufgd.edu.br (Willyam R.P Barros)

**Table S1.** Electrodeposition parameters of AuNPs.

| **Parameters** | **Cyclic voltammetry** | | **Chronoamperometry** | |  |
| --- | --- | --- | --- | --- | --- |
| Concentration (mmol L^-1^) | | 0.5 | | 0.5 | |
| Potential (V) | | -1.5 to +0.8 | | 0.22 | |
| Number of cycles | | 15 | | - | |
| Scan rate (mVs^-1^) | | 25 | | - | |
| Time (s) | | - | | 150 | |

**Table S2.** DPV parameters for UA determination.

| **Parameters** | **Tested intervals** | **UA** |
| --- | --- | --- |
| **Potential (V)** |  | 0.0 – +0.8 |
| **Step (V)** | 0.001 – 0.009 | 0.009 |
| **Modulation amplitude (V)** | 0.01 – 0.09 | 0.05 |
| **Modulation time (s)** | 0.01 – 0.09 | 0.03 |
| **Interval time (s)** | 0.1 – 0.9 | 0.8 |
| **Electrolyte** |  | PBS 0.1 mol L^-1^  (pH 7.0) |

**Table S3.** EDX elemental mapping.

| **Element** | **Atom (%)** |
| --- | --- |
| Carbon | 95.82 |
| Oxygen | 0.87 |
| Gold | 0.49 |
| Nitrogen | 2.48 |
| Bromine | 0.34 |

**Figure S1.** Au electrodeposition profiles for A) different chronoamperometry times and B) 15 cycles of CV.

**A**

**B**

**Figure S2.** DPV profiles for the optimization of SPE/Au/g-C_3_N_4_@CTAB (0.5 mmol L^-1^ Au electrodeposited by chronoamperometry) for 200 µmol L^-1^ UA in 0.1 mol L^-1^ PBS at pH 7.0. **A)** Different concentrations of g-C_3_N_4_ (0.5, 1.0, 2.0 mg mL^-1^) with fixated CTAB concentration at 5.0 mmol L^-1^ and **B)** varying concentration of CTAB with fixated g-C_3_N_4_ concentration fixated at 0.5 mg mL^-1^.

**A**

**B**


**Figure S3.** Optimization of Au electrodeposition (**A**) different chronoamperometry deposition times and (**B**) DPV profiles comparison between CV and Chronoamperometry electrodeposition for 200 µmol L^-1^ UA.

**A**

**B**

**Figure S4.** EDX spectra.


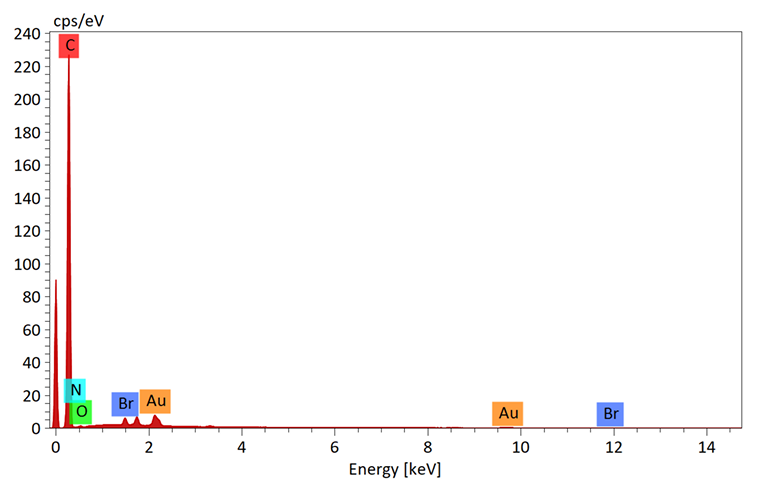

**Figure S5.** CV voltammograms varying scan rate from 5 to 150 mVs^-1^ for 0.1 mol L^-1^ KCl in the presence of 1.0 mmol L^-1^ [Fe(CN)_6_]^3-^/[Fe(CN)_6_]^4^. **A)** SPE/Au, **B)** SPE/Au/g-C_3_N_4_, **C)** SPE/Au/g-C_3_N_4_@CTAB and **D)** linear relation between current response and scan rate.

**C**

**B**

**A**

**D**

**Figure S6.** Reproducibility analysis across five different electrodes using DPV for 200 µmol L^-1^ UA at 0.1 mol L^-1^ PBS pH 7.0.

*RSD = 2.84%*
